# Mr. Towsey, on the Pernicious Effects of Ipecacuanha

**Published:** 1810-05

**Authors:** William Towsey

**Affiliations:** Lymington


### *To the Editors of the Medical and Physical Journal.*

GENTLEMEN,

As Dr. Hamilton, in the last Number of the Medical Journal, has requested communications from your Correspondents, relative to the pernicious effects of ipecacuanha, I am induced to send you the following fact.

Mrs. D——, a near relative of mine, and wife of Mr. T. D——, surgeon and apothecary, at Winborne in Dorsetshire (to whom I was an apprentice) was, during some years of the early part of her marriage, subject to most violent and frequent attacks of dyspnœa catarrhalis, which (not being relieved by any means that could be devised) rendered her situation truly miserable; at length Mr. D. acaccidentally met with an old magazine, in which a similar case of the wife of another medical man was recorded; and which was found to have been occasioned by the effluvia of ipecacuanha. It soon appeared (by experiment) that Mrs. D——'s complaint arose from the same cause; and from that time (the greatest care being taken in the use of the drug) she was freed from the disease; I however recollect, that frequently when it was used in the house, although she was at a distant part of it, she would be aware, from her sensation, of what was going on.

I am, &c. WILLIAM TOWSEY.

*Lymington, April* 9, 1810.

